# Neurofeedback Using Real-Time Near-Infrared Spectroscopy Enhances Motor Imagery Related Cortical Activation

**DOI:** 10.1371/journal.pone.0032234

**Published:** 2012-03-02

**Authors:** Masahito Mihara, Ichiro Miyai, Noriaki Hattori, Megumi Hatakenaka, Hajime Yagura, Teiji Kawano, Masaki Okibayashi, Nobuyoshi Danjo, Akihiro Ishikawa, Yoshihiro Inoue, Kisou Kubota

**Affiliations:** 1 Neurorehabilitation Research Institute, Morinomiya Hospital, Osaka, Japan; 2 Precursory Research for Embryonic Science and Technology (PRESTO), Japan Science and Technology Agency (JST), Saitama, Japan; 3 Department of Rehabilitation, Morinomiya Hospital, Osaka, Japan; 4 R&D Department, Medical Systems Division, Shimadzu Corp., Kyoto, Japan; Tokyo Metropolitan Institute of Medical Science, Japan

## Abstract

Accumulating evidence indicates that motor imagery and motor execution share common neural networks. Accordingly, mental practices in the form of motor imagery have been implemented in rehabilitation regimes of stroke patients with favorable results. Because direct monitoring of motor imagery is difficult, feedback of cortical activities related to motor imagery (neurofeedback) could help to enhance efficacy of mental practice with motor imagery. To determine the feasibility and efficacy of a real-time neurofeedback system mediated by near-infrared spectroscopy (NIRS), two separate experiments were performed. Experiment 1 was used in five subjects to evaluate whether real-time cortical oxygenated hemoglobin signal feedback during a motor execution task correlated with reference hemoglobin signals computed off-line. Results demonstrated that the NIRS-mediated neurofeedback system reliably detected oxygenated hemoglobin signal changes in real-time. In Experiment 2, 21 subjects performed motor imagery of finger movements with feedback from relevant cortical signals and irrelevant sham signals. Real neurofeedback induced significantly greater activation of the contralateral premotor cortex and greater self-assessment scores for kinesthetic motor imagery compared with sham feedback. These findings suggested the feasibility and potential effectiveness of a NIRS-mediated real-time neurofeedback system on performance of kinesthetic motor imagery. However, these results warrant further clinical trials to determine whether this system could enhance the effects of mental practice in stroke patients.

## Introduction

Motor imagery is a dynamic state during which a subject mentally simulates a specific movement without any overt movement [1]. There is ample evidence that motor imagery and motor execution share the same motor-related neural networks [2], [3], [4], [5], and several studies have shown that use of motor imagery can improve performance and learning in various motor tasks [6] with relevant cerebral reorganization [7]. Accordingly, mental practice with motor imagery has been introduced in the field of neurorehabilitation, although the efficacy of mental practice with motor imagery has been inconsistent. Several studies have revealed favorable improvements in motor outcomes after stroke [8], [9], [10], but insignificant effects have been also reported [11]. Several variables could be responsible for discrepancies in the utilization of imagery in a neurorehabilitation setting. First, direct monitoring for compliance during motor imagery is difficult, although several methods have been proposed for indirect monitoring of motor imagery, including use of autonomic nervous system responses [12] and test batteries [13], [14]. Second, recruited neural networks and training effects might depend on individual skill [4], [15] and method of motor imagery [5], [16], [17]. Motor imagery strategies can be characterized by kinesthetic motor imagery and visual motor imagery. During kinesthetic motor imagery, the subjects feel that they actually perform the movement with all the sensory consequences (first-person perspective). In contrast, during visual motor imagery, the subjects see themselves performing the movement as from a distance (third-person perspective). In a recent study, Stinear *et al.* showed that kinesthetic, not visual motor, imagery modulated motor cortical excitability, which suggested that kinesthetic motor imagery is more effective for motor learning than visual motor imagery [18]. Therefore, the inappropriate use of motor imagery could be one of the possible reasons why only a limited number of patients benefit from mental practice using motor imagery. Under this assumption, it was hypothesized that the efficacy of mental practice with motor imagery could be improved if the subjects performed appropriate mental imagery.

Previous neuroimaging studies have revealed that several cortical areas, including the premotor area, sensorimotor cortex, and inferior parietal area, were more greatly activated using kinesthetic (first-person perspective) motor imagery compared with visual (third-person perspective) motor imagery [5], [16]. Based on these findings, activation feedback of motor-related cortical areas to subjects (neurofeedback) could augment the quality and skill of motor imagery.

Although the concept of neurofeedback is not novel, the technique has recently attracted a great deal of attention with regard to a “brain-computer interface” [19], [20]. Several candidates for use in a neurofeedback system exist among various neuroimaging modalities. In a clinical setting, studies using an electroencephalography (EEG)-mediated system have reported that real-time EEG feedback enables voluntary regulation of cortical activation and attentional levels [21], [22], and such feedback is effective for treating attention deficit and hyperactivity disorder [23], as well as epilepsy [24]. Several reports have demonstrated the effectiveness of fMRI, which exhibits excellent spatial resolution of neurofeedback activities with real-time data processing [25], [26]. Voluntary regulation of emotion-related brain activities [27], [28] and enhancement of regional brain activities during motor execution [29] have been reported using an fMRI-mediated neurofeedback. However, despite promising findings using fMRI-mediated neurofeedback systems, the relatively large-scale equipment requirements and strict subject constraints could serve as drawbacks when applying neurofeedback-based training in clinical settings, including rehabilitation medicine.

The near-infrared spectroscopy (NIRS) system, which is another neurofeedback system candidate, could be useful in a clinical setting, because NIRS noninvasively measures regional hemodynamic changes in oxygenated and deoxygenated hemoglobin (OxyHb and DeoxyHb) associated with neuronal activation [30], [31]. Moreover, NIRS is relatively robust with regard to subject motion, and relatively little time is needed for attachment without paste, which leads to less onerous constraints. For the present study, a NIRS-mediated neurofeedback system was developed, which included online and real-time processing of task-related hemoglobin signal changes in visual feedback of subjects.

The first aim of the study was to determine whether the system reliably estimated task-related cortical activation. Subsequently, whether neurofeedback could enhance cortical activation associated with motor imagery was analyzed. Sequential finger movements from the right hand were utilized for the motor imagery task; hemoglobin signal changes from the left motor cortex were evaluated as “real” feedback, and sham information irrelevant to cortical signals served as control or “sham” feedback. A small feasibility study was initially conducted using actual finger movements for the task. The main purpose of this initial experiment was to confirm that the real-time signal analysis method was consistent with off-line analyses, as well as to determine which hemoglobin oxygenation parameters – Oxy- or Deoxy-Hb signals – would be suitable for the feedback signal. Based on findings from the first experiment, a second experiment was conducted in which self-assessment scales of motor imagery and cortical activation mapping were compared between the two feedback conditions to determine whether real feedback significantly affected motor imagery quality and related cortical activation.

## Methods

### Ethical Statement

In accordance with the Declaration of Helsinki, written informed consent was obtained from each subject, who participated in the present study. The study was approved by the Ethics Committee of Morinomiya Hospital (Osaka, Japan).

### Experiment 1

#### Subjects

A total of five healthy, right-handed subjects were recruited to test consistency of the neurofeedback system. Handedness of the subjects was measured by the self-report of the side of their hand used in writing and eating, and no subjects had history of correction of handedness. The mean (± SD) age of participants was 36.8 (±13.5) years, with one female subject. Written informed consent was obtained from each subject.

#### Task

**Figure 1:**
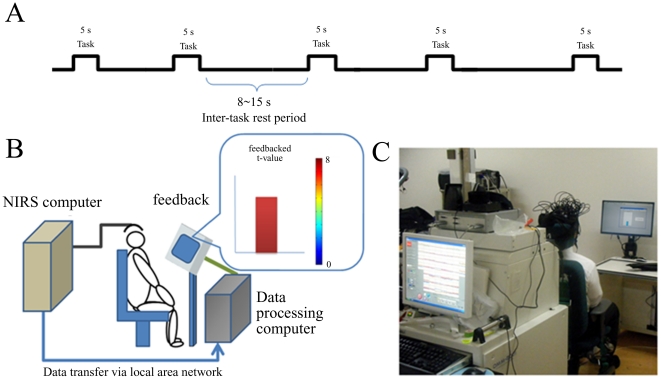
Configuration and testing of the neurofeedback system using NIRS. ***A,*** Representation of time course of the experiment. Subjects were asked to perform 15 repetitions of a 5-s task with randomized inter-task rest periods between 8–15 s. The total length of one experimental session was no longer than 250 s. ***B,*** Schematic figure of the NIRS-mediated neurofeedback system. Task-related cortical hemoglobin signal changes were transferred to a data-processing computer, and the evaluated cortical activation was visually fed back in real-time. Cortical activation was represented by bar height and color. ***C,*** The NIRS-mediated neurofeedback system in use. Subjects were seated in an armchair, and the heads were fixed to the headrest to avoid excessive head movement during experimentation.

Self-paced, sequential movements of the right fingers were used for the motor task. Participants were asked to sequentially fold their right fingers from the thumb to the little finger, and then to unfold them from the little finger to the thumb. They repeated these movements during the 5-s task period. The experiment consisted of 15 executions of the motor task, with randomized inter-task rest periods ranging from 8–15 s (Figure 1A). The total time length of experiment was no longer than 250 s. During the experiment, subjects sat comfortably in an armchair with a headrest, with their arms on the armrests. To avoid excessive head movements, the head was fixed to the headrest with an elastic band.

#### NIRS-mediated neurofeedback system

The NIRS-mediated neurofeedback system consisted of the NIRS system, a data-processing computer, and a monitor to display feedback information. A schematic overview of this system is illustrated in Figure 1B, and Figure 1C shows the system in use. To detect task-related hemoglobin signal changes, a continuous-wave NIRS system (OMM-3000, Shimadzu Corp., Kyoto, Japan) with 16 light emitter fibers and 16 light detector fibers was employed.

It was assumed that NIRS detects hemoglobin signal changes derived from local vascular reactions coupled with neuronal activation at the cortical surface [32], [33], [34]. In the present study, 5-ms pulses of near-infrared light at wavelengths of 780 nm, 805 nm, and 830 nm were emitted from each of the emitter fibers, respectively [35], [36], [37]. Emitted light was absorbed by OxyHb and DeoxyHb and attenuated by scattering in tissues, which was detected by a detector fiber located 3 cm from each emitter fiber. OxyHb and DeoxyHb signal changes were calculated according to the modified Beer-Lambert Law for highly scattering media [38]. For each wavelength, absorbance at the start of measurement was defined as the initial absorbance. Because it was not possible to measure the differential path-length factor using the continuous-wave NIRS system, it was assumed that it was constant, and hemoglobin signal changes were denoted in arbitrary units of millimolar-millimeter (mM×mm) [39].

**Figure 2:**
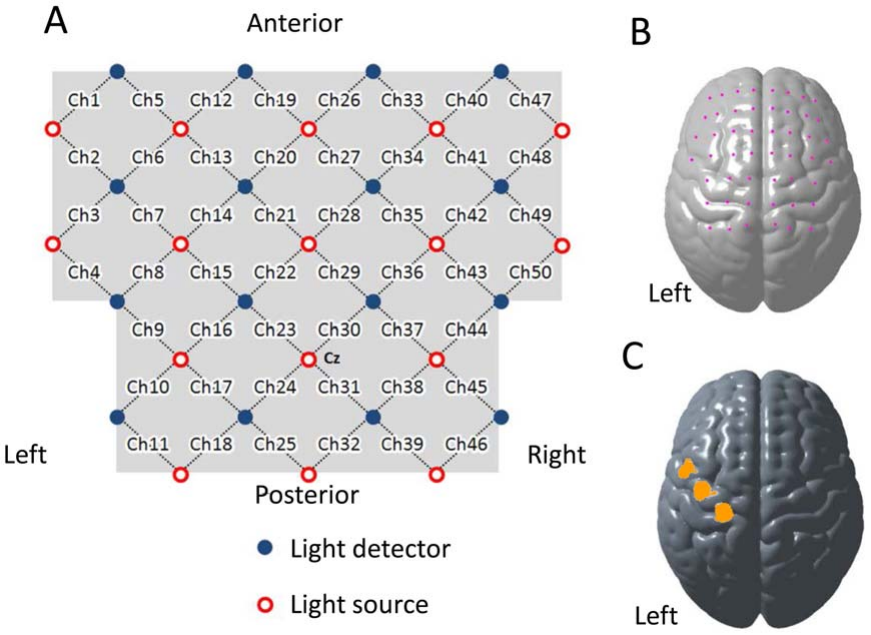
Cortical placement of NIRS channels. ***A,*** Fiber arrangement for the 50-channel NIRS system. The light source at the center of the third row was placed at the subject's vertex (Cz). ***B,*** Estimated location of each NIRS channel, which was defined as the midpoint of the line between the corresponding light source-detector pair. ***C,*** Estimated cortical area covered by channels 4, 9, and 17, which was set as the neurofeedback source of cortical activation related to the motor imagery task.

According to the fiber arrangement shown in Figure 2, 50-channel measurements of hemoglobin signal changes from the frontoparietal skull surface were performed. As described in our previous study [35], a custom-made, hard-plastic cap, with an inter-optode distance of 3.0 cm, was used to hold the fibers tightly to the skull surface. For each subject, total experimental time using the NIRS system was not longer than 15 min. A light source at the center of the third row served as the anchor point and was placed at the subject's vertex (Cz). It was assumed that head sizes and shapes were comparable, because the hard-plastic cap fit well on all participants. Using this fiber arrangement, the C3 position was placed between the light detector at the leftmost of the third row and the light source at the leftmost of the third row (area of channel 9 in the Figure 2) in all subjects. Because the international 10–20 standard positions exhibit a certain level of standard deviation [40], and the NIRS system results in relatively low spatial resolution due to the banana-shaped propagation path of detected signals [41], it was assumed that the cortical location of each channel was comparable among participants. Therefore, the approximate cortical location of each channel was estimated from anatomical MRI data of representative subjects. Similar to our previous study [35], 3D T1-weighted MRI scans were obtained from two subjects, and the optode location was marked with a 3D digitizer (FASTRAK; Polhemus, Colchester, VT). After calculating the midpoint of the neighboring light source and detector on the skull surface, the fNIRS channel locations on the cortex were estimated using the balloon-inflation method [40]. Anatomical normalization to the Montreal Neurological Institute (MNI) standard template [42] was performed using 12-parameter affine transformation. In addition, the cortical region covered by each channel was estimated using MRIcro software (by Chris Rodan: http://www.MRIcro.com), together with the Brodmann's area (BA) image and Automated Anatomical Labeling (AAL) image [43], which were downloaded from the website. Because the pre-segmented template images were aligned with normalized brain images in the MNI coordinate system, it was possible to estimate cortical regions and BA covered by each channel. Results from two representative subjects were comparable.

#### Data processing

NIRS measures task-related changes in OxyHb and DeoxyHb signals on the cortical surface. In the present study, the OxyHb signal was primarily utilized as a cortical activation marker and feedback signal source. Previous studies demonstrated that OxyHb signals exhibit superior sensitivity in task-related signal changes and a greater correlation with blood oxygen level–dependent signals in functional magnetic resonance imaging (fMRI) [34], [44]. However, DeoxyHb signals were also analyzed to determine the most appropriate hemoglobin parameter. Hemoglobin signals were measured at a sampling rate of 4 Hz, and these data were processed in the NIRS computer and transferred to a data-processing computer *via* a local area network cable. In the data-processing computer (Endeavor Pro 7000, Seiko Epson Corp, Japan), transferred data were buffered and processed using a general linear model (GLM) and least-square estimation, which can be suitable for using shorter inter-task intervals [45]. Statistical evaluations of real-time estimation of cortical hemoglobin signal changes were performed with MATLAB software (ver. 7.10, Mathworks, Natick, MA).

**Figure 3:**
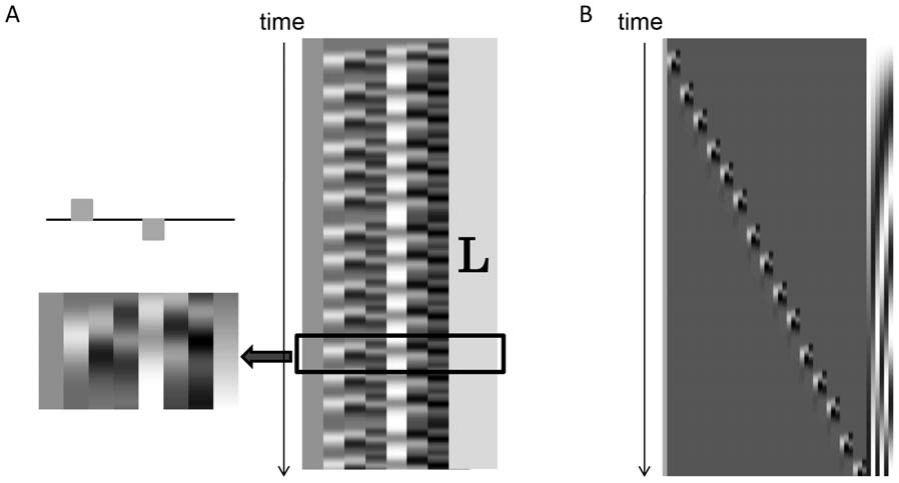
Design matrices for real-time processing and off-line processing. ***A,*** The design matrix for real-time sliding-window GLM analysis. The time window was 80 data points wide. The matrix consisted of one constant column, three columns for task and rest phases, respectively, and one linear term (L). Task-related signal changes were estimated as a beta value, comparing task data with resting data. ***B,*** The design matrix for off-line task-by-task GLM analysis. The matrix consisted of a constant column, columns for each task, and a high-pass filter with a cut-off frequency of 0.0125 Hz.

A two-parameter gamma hemodynamic response function (HRF), which was utilized in fMRI data analysis, served as the predictor for task-related hemoglobin signal changes [46]. Temporal and dispersion derivatives were included to modulate HRF onset and dispersion. A sliding-windows GLM analysis with least-squares estimation was utilized for real-time analysis of signal changes. The observation window was 80 data points wide and covered at least one task block; each observation window was measured for 20 s at 4 Hz. The design matrix for estimating task-related hemoglobin signal changes comprised eight columns: a constant column for collecting offsets, three columns containing box-car functions for the 5-s task phase convolved with the three basis sets for HRF, three columns containing box-car functions for resting phase observations convolved with three basis sets for HRF, and a linear term for correcting linear drift (Figure 3A). To evaluate cortical activation of each channel, estimated beta values of the task phase (the second column) were compared with the rest phase (the fifth column). To adjust for auto-correlated error terms, a autoregressive model of order 1 was used [46]. The contrasted beta-value was evaluated using the one-tailed one-sample *t*-test against zero, and the calculated *t*-value was used as a marker of cortical activation at each channel. Because it took 50 ms to calculate *t*-values for 80 points of data from 50 NIRS channels, it was possible to calculate *t*-values for each data acquisition point. As shown in Figure 2C, channels 4, 9, and 17 were thought to cover the left sensorimotor and adjacent motor-related cortex, and the maximal calculated *t*-value for these three channels is displayed as the height and color of the vertical bar to provide feedback for the subjects (Figure 1). If all *t*-values from the three channels were negative, no significant cortical activation was considered to have occurred, and the feedback bar was set to zero. Because the target t-values for significant task-related cortical activation were set to >2.0 (approximately indicates *P*<0.05), the maximum height of the vertical bar was set to 8.0 for better visibility. The displayed vertical bar color, which ranged from blue (zero) to red (8.0), also varied according to the *t*-value.

#### Test for real-time assessment of cortical activation

As a reference standard for cortical activation of each task, an off-line task-by-task GLM analysis was performed using all data points for the dataset. In this analysis, the time series was divided by task, and each individual task was represented by three box-car functions together with three basis sets for HRF. The design matrix consisted of 52 columns: a constant for collecting offsets, 3×15 columns (3 basis functions×15 repetitions of the task) for task data, and 6 columns for discrete cosine transform functions as high-pass filters with a cut-off frequency of 0.0125 Hz to remove baseline drifts (Figure 3B). The *t*-values were calculated for each task/channel, and maximal *t*-values in channels 4, 9, and 17 were used as reference standards for cortical signals for each task (T_Ref_).

To analyze real-time assessment, the referenced cortical signals calculated from the task-by-task analysis were compared with feedback signals calculated from the sliding-window GLM analysis. By comparing reference signals, *t*-values were calculated from the sliding-window GLM analyses and were averaged from onset of one task to the next. The averaged value (T_SWA_) served as the feedback signal for each task. Although OxyHb signal-based analysis was most often utilized, a similar correlation analysis was performed using DeoxyHb signals. Pearson's correlation coefficient was used to measure correlations between the two cortical signals from each subject. In addition to the level of significance (*P*<0.05), r-values were calculated for effect size and represented small (0.1<), medium (0.3<), or large (0.5<) correlations [47].

### Experiment 2

#### Subjects

A total of 24 healthy, right-handed subjects were recruited, with no history of neurological or psychological disease. As Experiment 1, handedness of the subjects was measured by the self-report of the side of their hand used in writing and eating, and no subjects had history of correction of handedness. Written informed consent was obtained from each subject. Only two subjects, who participated in Experiment 1, were included.

#### Task

Participants were asked to perform two sessions of the motor imagery task. Each session consisted of 15 sets in which participants performed imagery of right-finger movements, without physical movement, for 5 s. Environmental settings were similar to those in Experiment 1. Subjects were asked to imagine self-paced and sequential folding of the right fingers similar to the movements in Experiment 1. The subjects were also asked to kinesthetically imagine movements rather than visually (*e.g.*, feel the movements as they physically perform the task) [5]. To ensure task consistency among subjects, several minutes of pre-training were required prior to experimentation. In the pre-training session, subjects performed physical finger movement under similar experimental settings, including feedback. During the finger movement task, finger movement was visually inspected and, if needed, the subjects were asked to move their fingers at a constant pace. They were also instructed that the vertical bar would be higher if kinesthetic imagery was performed better during the task period and the subjects were more relaxed during rest periods. After training of the physical finger movement task, several minutes of motor imagery without feedback practice were also prescribed. Subjects were asked to imagine constant finger movement similar to what they had physically performed. Throughout the experiment, finger movements were visually inspected. Three subjects were excluded due to overt finger movements during the motor imagery task. The remaining subjects did not make finger movement during the imagery task. The total length of each motor imagery session was no longer than 250 s, and the length of the rest interval between each imagery task was randomized from 8–15 s. Participants were also allowed several minutes of rest between the two imagery sessions to avoid fatigue and concentration loss.

During the motor imagery task, participants were instructed to watch the monitor where feedback information was displayed as vertical bar height and color (Figure 1). In one session of the motor imagery tasks (real feedback session), *t* -values derived from hemoglobin signals in the left (contralateral) motor cortex (Ch. 4, 9, and 17) were displayed as vertical bars. Based on results from Experiment 1, OxyHb signal changes served as measurements of cortical activation for feedback. In the sham feedback session, normalized random values irrelevant to cortical activation were used for feedback, similar to a previously described neurofeedback study using functional MRI [29]. Sham feedback values with a mean value of 1.2 and standard deviation of 0.3 were generated by the MATLAB function *randn*. The generated values were truncated if values were <0. The subjects were aware that only one of the two experimental sessions was real and the other was a sham condition. However, the order of real and sham feedback sessions was randomized and this information was not provided to the subjects.

After each session, the participants were asked to evaluate a self-assessment scale of motor imagery quality. They were asked to image finger-folding and to score how well they kinesthetically imagined the finger movements. If the subject experienced a vivid kinesthetic feeling that he/she had performed the task physically, then the 11-point scale scored a performance of 10, while the worst performance was scored as zero.

#### Data analyses

To estimate the effect of neurofeedback on motor imagery quality, self-assessment scale scores under real and sham feedback conditions were compared using a two-tailed paired *t*-test. To exclude the possibility that the order of motor imagery feedback conditions affected motor imagery quality, self-assessment scales were compared between first and second sessions in order. In addition, the average height of the presented feedback bar was compared between the two conditions, because it could possibly influence the self-assessment scores. Pearson's correlation analysis was used to compare self-assessment scores and presented height of feedback bar. The significance level was set to *P*<0.05.

As a first-level analysis, the effect of neurofeedback on cortical activation maps associated with motor imagery was analyzed, and the contrast between motor imagery task and baseline in real and sham feedback conditions was estimated. In addition, intra-subject contrasts between the two conditions were evaluated. Accordingly, three beta-values were calculated from four different contrasts, including real feedback *vs.* baseline, sham feedback *vs.* baseline, sham *vs.* real, and real *vs.* sham.

Oxy- and DeoxyHb signal changes were analyzed. For each contrast, a positive beta-value indicated an increase, and a negative beta value indicated a decrease in hemoglobin signals in the former condition compared to the latter condition. In the design matrix, discrete cosine transform functions were included as high-pass filters with a cut-off frequency of 0.0125 Hz to remove baseline drifts. Averaged signal changes from 50 channels were included for eliminating global effects, such as autonomic responses relevant to motor imagery. To adjust for the auto-correlated error term, an autoregressive model of order 1 was used [46]. As a second-level analysis, a random-effect analysis [48], based on beta weight of each subject, as the dataset was performed; one-tailed one-sample *t*-test distinct from zero was performed for the contrast between task and baseline, and two-tailed one-sample *t*-test was performed for the contrast between two feedback conditions. The significance level was set to *P*<0.01 (uncorrected).

In addition to statistical analysis using GLM, a timeline analysis of OxyHb and DeoxyHb signals from the left lateral premotor cortex (channels 3), left sensorimotor cortex (channel 4), and left parietal association cortex (channel 25) was also performed. In each channel, averaged data from 315 trials (15 trials×21 subjects) under real feedback and sham feedback conditions were plotted from 1 s before to 12 s after task onset to validate GLM analysis.

## Results

### Experiment 1

**Figure 4:**
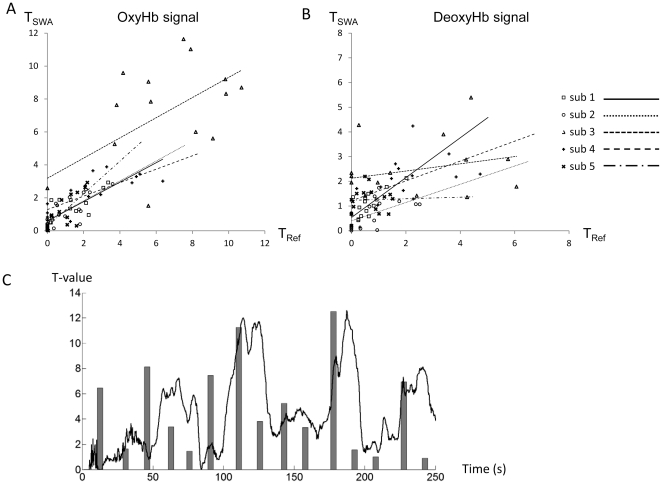
Comparison of calculated *t*-values from real-time processing and off-line processing. ***A, B,*** Scatter plot of calculated *t*-values from real-time processing and off-line processing in five subjects using (B) OxyHb signal data and (C) DeoxyHb signal data. Using OxyHb signal data, all five subjects exhibited significant correlations between real-time assessments of cortical activation calculated from sliding-window GLM analysis (T_SWA_) and reference cortical activation calculated from task-by-task GLM analysis (T_Ref_). However, correlations between T_SWA_ and T_Ref_ were less with DeoxyHb data. ***C,*** The dynamic change of cortical activation feedback, as calculated from sliding-window GLM analysis (black line) and reference cortical activations calculated from the task-by-task GLM analysis (gray bar) in Experiment 1 (data from a representative subject).

In the feasibility study, which utilized NIRS-mediated real-time neurofeedback system data from five healthy right-handed subjects, all subjects completed the right-finger folding task without obvious head motion. Correlation analyses between real-time feedback signals derived from sliding-window GLM analyses (T_SWA_) and results from conventional task-by-task GLM analyses (T_Ref_) in five subjects are shown in Table 1. Significant and positive correlations were revealed in all subjects using OxyHb data (Figure 4A), but DeoxyHb signals resulted in correlations (Figure 4B) with lower r-values. The r-values from two different hemoglobin signals were compared, demonstrating that OxyHb signals were statistically more robust in the current neurofeedback system [47]. Therefore, the OxyHb signal-based feedback was used for subsequent experiments. The representative time course of real-time feedback signals, as well as results from the conventional task-by-task GLM analyses using OxyHb signals, are shown in Figure 4C. Results demonstrated that cortical activations varied task-by-task, and real-time analysis evaluated task-related cortical activation with a several-second delay. There was no uniform trend for the task-related cortical activation change among subjects.

**Table 1:** Correlation analysis between t-values calculated from task-by-task analyses (T_Ref_) and t-values calculated from sliding-window GLM analyses (T_SWA_).

Subject	Correlation coefficient	p-value
**A: Correlation coefficients using OxyHb data**		
**1**	**r = 0.7827**	**p<0.001**
**2**	**r = 0.7206**	**p<0.005**
**3**	**r = 0.6045**	**p<0.05**
**4**	**r = 0.7601**	**p<0.005**
**5**	**r = 0.7662**	**p<0.001**
**B: Correlation coefficients using DeoxyHb data**		
**1**	**r = 0.7617**	**p<0.005**
**2**	**r = 0.5559**	**p<0.05**
3	r = 0.262	p = 0.3455
**4**	**r = 0.5441**	**p<0.05**
5	r = 0.0328	p = 0.9075

### Experiment 2

**Figure 5:**
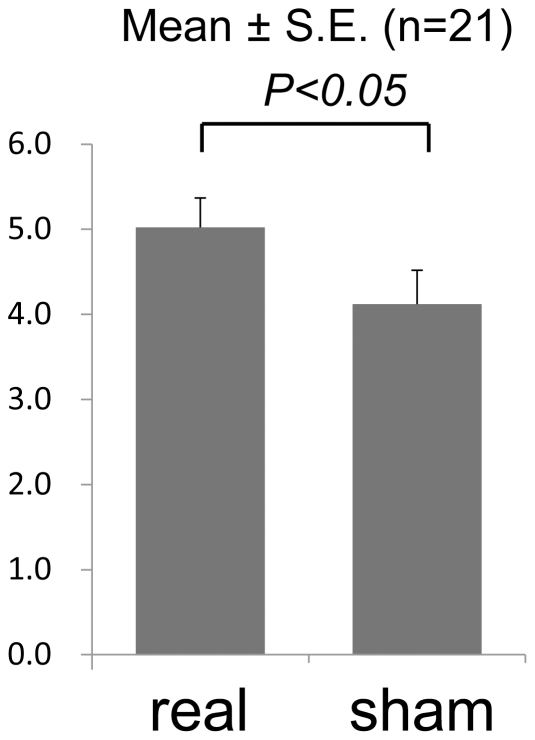
Self-assessment scale scores for kinesthetic motor imagery under real and sham feedback conditions. Paired *t*-test revealed increased self-assessment scores for kinesthetic motor imagery under real feedback conditions.

Although 24 healthy, right-handed subjects were recruited to analyze the effects of neurofeedback on motor imagery task-related cortical activation, data from three subjects were excluded due to overt finger movements during the motor imagery task. Subsequent analyses comprised data from the remaining 21 subjects. The mean (± SD) age of the 21 subjects was 34.3 (±10.3), with 4 female subjects. The average (± SD) self-assessment scale scores for kinesthetic motor imagery, which were assessed after real- and sham-feedback conditions, were 5.0 (±1.6) and 4.1 (±1.8), respectively (Table 2). The scores were significantly greater under real feedback conditions (Figure 5, t_20_ = 2.53, *P*<0.05), but the order of conditions did not affect the score (t_20_ = 1.89, *P* = 0.07). The mean (± SD) heights of presented feedback bars under real and sham feedback conditions were 1.7 (±0.6) and 1.2 (±0.03), respectively, and there was a significant difference between the conditions (t_20_ = 4.37, *P*<0.001). However, the correlation between presented feedback bar height and self-assessment scores was small and non-significant (r = 0.195, *P*>0.05). Most subjects were not aware of the order of the two conditions.

**Table 2:** Individual self-assessment scores from 21 participants.

			Self assessment for motor imagery		Average feedback presentation		
Subject	Gender	Age	Real	Sham	Real	Sham	Order of real feedback
1	F	24	5	8	1.31±0.67	1.13±0.29	First
2	M	45	4	3	1.38±0.88	1.11±0.28	Second
3	F	37	5	4	1.73±1.41	1.18±0.27	First
4	M	47	5	3	2.68±1.96	1.17±0.27	Second
5	M	24	4	3	1.79±0.99	1.19±0.30	First
6	M	29	6	2	1.67±0.98	1.18±0.27	Second
7	M	42	2	1	1.22±0.67	1.18±0.24	First
8	M	48	6	5	0.88±1.69	1.21±0.26	Second
9	M	39	6.5	5	3.12±3.12	1.11±0.27	First
10	M	49	5	6	1.28±0.73	1.14±0.25	Second
11	M	35	4	6	1.31±0.67	1.18±0.26	First
12	M	24	6.5	3.5	0.97±0.76	1.11±0.28	Second
13	F	24	2	1	1.42±1.36	1.13±0.27	First
14	M	27	7	5	1.63±0.76	1.16±0.28	Second
15	M	38	5	4	2.01±2.00	1.11±0.30	First
16	M	55	4	2	2.38±1.82	1.11±0.28	Second
17	M	23	7	6	1.38±0.88	1.17±0.26	First
18	M	36	3	3	1.92±1.48	1.18±0.26	Second
19	M	26	5	5	1.88±0.84	1.16±0.29	First
20	M	25	8	5	1.58±0.90	1.11±0.27	Second
21	F	23	5.5	6	1.26±0.91	1.20±0.25	First

M: male F: female.

**Figure 6:**
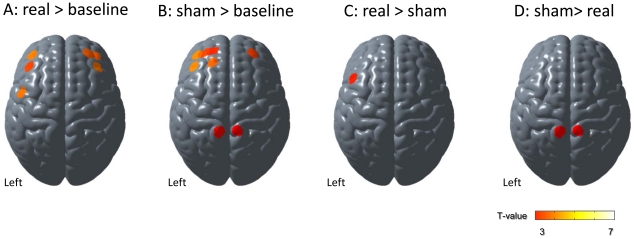
Cortical mapping of motor imagery–related activation. Results from second-level random effect analysis of comparisons between real feedbacks *vs.* baseline *(A)*, between sham feedback *vs.* baseline *(B)*, real *vs.* sham feedback *(C)*, and sham *vs.* real feedback *(D)*. Within-subject comparison between feedback conditions revealed significantly increased cortical activation in the left lateral premotor cortex under real feedback conditions compared with sham feedback conditions, as well as significantly increased activation in the bilateral parietal association cortex under sham feedback conditions compared with real feedback conditions.

Cortical activation mapping with OxyHb signals revealed significantly increased motor imagery-related signals in the left sensorimotor and bilateral prefrontal cortex under real feedback conditions (Figure 6A, and Table 3A). Under sham feedback conditions, motor imagery-evoked signals resulted in significantly increased cortical OxyHb signals in the bilateral prefrontal and bilateral parietal association cortex, although sensorimotor activation did not reach statistical significance (Figure 6B, and Table 3B). Comparison of motor imagery-related cortical activation between two different feedback conditions revealed significantly increased OxyHb signals in the left lateral premotor cortex under real feedback conditions (Figure 6C, and Table 3C). Compared with real feedback conditions, the bilateral parietal association cortex exhibited significantly increased OxyHb signals under sham feedback conditions(Figure 6D, and Table 3D). In analyses with DeoxyHb signals as the measure of cortical activation, more limited areas were significant (Table 4). The left parietal association cortex exhibited significantly decreased DeoxyHb signals under sham feedback conditions compared to baseline. Comparison of motor imagery-related cortical activation between two feedback conditions also revealed significantly decreased DeoxyHb signals in the left parietal association cortex under sham feedback conditions. However, DeoxyHb signals were not significantly decreased under real feedback conditions compared with the baseline.

**Table 3:** OxyHb signal-based cortical mapping analysis for motor imagery with feedback.

CH	MNI coordinates (X/Y/Z)	Cortical region	BA	*t*-value
***A: Activated cortical regions under the real feedback condition (comparisons between real feedback vs. baseline)***				
Left sensorimotor cortex				
4	−47/−8/57	PreCG	4/6	3.70
Left prefrontal cortex				
1	−38/51/30	MFG	46	3.83
2	−45/36/43	MFG	9/46	3.23
Right prefrontal cortex				
47	41/47/29	MFG	46	3.53
48	45/28/42	MFG	9/46	3.80
40	31/51/35	MFG	9/46	3.54
***B: Activated cortical region under the sham feedback condition (comparisons between sham feedback vs. baseline)***				
Left prefrontal cortex				
1	−38/51/30	MFG	46	3.89
2	−45/36/43	MFG	9/46	3.87
5	−26/56/35	MFG	9/46	3.28
12	−19/57/37	SFG	9	2.98
13	−18/40/50	SFG	9	3.54
Left parietal association cortex				
25	−9/−61/72	Precuneus/SPL	5/7	2.67
Right prefrontal cortex				
40	31/51/35	MFG	9/46	3.26
Right parietal association cortex				
32	6/−61/70	Precuneus/SPL	5/7	2.55
***C: Enhanced cortical regions under the real feedback compared with the sham feedback condition (comparisons between real vs. sham feedbacks)***				
Left premotor cortex				
3	−46/11/53	MFG	6	2.93
***D: Enhanced cortical regions under the sham feedback compared with the real feedback condition (comparisons between sham vs. real feedbacks)***				
Left parietal association cortex				
25	−9/−61/72	Precuneus/SPL	5/7	2.60
Right parietal association cortex				
32	6/−61/70	Precuneus/SPL	5/7	2.55

CH: channel number; BA: Brodmann area; PreCG: precentral gyrus; SFG: superior frontal gyrus; MFG: middle frontal gyrus; SMA: supplementary motor area; SPL: superior parietal lobule.

**Table 4:** DeoxyHb signal-based cortical mapping analysis for motor imagery with feedback.

CH	MNI coordinates (X/Y/Z)	Cortical region	BA	*t*-value
***A: Activated cortical region under the sham feedback condition (comparisons between sham feedback vs. baseline)***				
Left parietal association cortex				
25	−9/−61/72	Precuneus/SPL	5/7	2.90
***B: Enhanced cortical regions under the sham feedback compared with the real feedback condition (comparisons between sham vs. real feedbacks)***				
Left parietal association cortex				
25	−9/−61/72	Precuneus/SPL	5/7	3.06

CH: channel number; BA: Brodmann area; SPL: superior parietal lobule.

**Figure 7:**
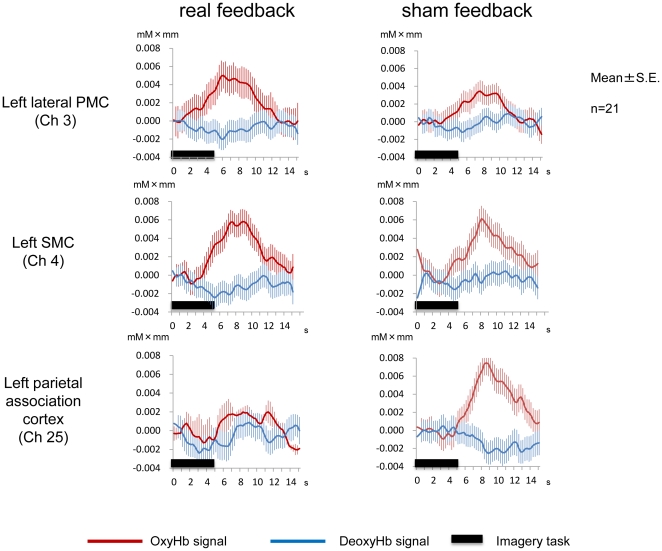
Average hemoglobin signal changes in the left SMC, left PMC, and left parietal association cortex. In the left PMC, task-related OxyHb signal changes increased under real feedback conditions. In the left parietal association cortex, task-related OxyHb signal changes increased under sham feedback conditions. OxyHb signal changes were comparable between feedback conditions in the left SMC. In the left parietal association cortex, task-related DeoxyHb signals decreased only under sham feedback conditions.

Figure 7 shows average time courses of OxyHb and DeoxyHb signal changes from the left sensorimotor cortex (channel 4), left lateral premotor cortex (channel 3), and left parietal association cortex (channel 25) in all 21 participants. In the left sensorimotor cortex, task-related OxyHb signal changes were comparable between real and sham feedback conditions (t_20_ = 1.13, *P* = 0.14). However, in the left lateral premotor cortex, task-related OxyHb signals were more evident under real feedback conditions compared with sham feedback conditions (t_20_ = 2.93, *P*<0.005). In contrast, the bilateral parietal association cortex exhibited significantly increased OxyHb signals under sham feedback conditions compared with real feedback conditions (t_20_ = 2.60, *P*<0.01). The task-related DeoxyHb signal did not significantly change in the left lateral premotor and sensorimotor cortex (t_20_ = 0.99, *P* = 0.17, t_20_ = 1.13, *P* = 0.14, respectively), but decreased DeoxyHb signals in the left parietal association cortex were more evident under sham feedback conditions than real feedback conditions (t_20_ = 3.06, *P*<0.005). R from time-line analyses were consistent with findings from cortical mapping analyses, which suggested that neurofeedback induced enhanced contralateral premotor activation and reduced parietal association cortex activation.

## Discussion

Results from the present study demonstrated that the NIRS system can be used to detect real-time task-related hemoglobin signal changes, and this system can be reliably used as a neurofeedback tool. During motor learning processes, correct information feedback about performance (“knowledge of result”) is known to be effective in healthy subjects and in stroke patients [49], [50]. For tasks related to motor imagery, difficulty of objective evaluation has traditionally hampered collection and dissemination of correct information pertaining to task performance. However, results from the present study suggested that neurofeedback methods could provide important information about local brain activity associated with motor imagery.

Results from Experiment 1 revealed that hemoglobin signal changes were detected by the sliding-window GLM analysis with a hemodynamic delay of several seconds. Results from real-time processing in this system represented task-related cortical hemoglobin signal changes. Compared with other neuroimaging modalities, the NIRS-mediated neurofeedback system exhibits several advantages for clinical application, including relative robustness of subject motion, shorter attachment time, and less subject constraint. However, one main technical flaw of the NIRS measurements is the delay of several seconds between neuronal activation and hemoglobin signal changes. Simultaneous measurement of NIRS and EEG, which measures direct neuronal activation and has greater temporal resolution, could be a possible solution for methodological limitations [51], because these techniques produce a complementary effect. However, the combination of EEG and NIRS requires longer experimentation time and could reduce feasibility in a clinical setting.

The present results demonstrated that OxyHb was more robust under real-time assessment conditions for task-related cortical activation. However, it remains to be determined which hemoglobin parameters are more suitable for measuring cortical activation. Although the current theoretical framework for blood level-dependent (BOLD) signals in fMRI suggests that decreased DeoxyHb concentrations correlate with greater BOLD signals [52], some studies have reported greater correlations between BOLD and OxyHb signals [34], [44]. OxyHb signals have also been shown to be sensitive to changes, but DeoxyHb signals are more selective and localized [53]. Lower sensitivity and greater spatial selectivity for DeoxyHb signal changes could explain the present results. In addition, wavelength selection could affect sensitivity of Oxy- and DeoxyHb signals. The NIRS system utilized three wavelengths (780 nm, 805 nm, and 830 nm). However, several studies have reported that the 782–830 nm pair results in less SNR than the 692–830 nm pair [54]. In addition, the 790–825 nm pair results in less separation of OxyHb and DeoxyHb signals compared to the 710–905 nm pair [55]. These results suggest that wavelength selection in the current system could result in reduced sensitivity for DeoxyHb signal changes.

Results from Experiment 2 demonstrated that NIRS-mediated neurofeedback enhanced motor imagery-related cortical activation in the contralateral premotor cortex. These findings were consistent with previous findings from a fMRI-mediated neurofeedback study, which showed rostral enlargement of motor imagery-related motor cortical activation [29]. Other studies have also suggested that the premotor cortex is a crucial region for the generation of motor imagery [3], [56], and subjects with good motor imagery skills exhibit enhanced activation in the lateral premotor cortex compared to those with poor motor imagery skills [4]. These results suggest that the premotor cortex could be a candidate for neurofeedback information in the motor imagery task. Further studies are needed to determine which cortical area is most effective as a neurofeedback for motor imagery augmentation.

Results from the present study also demonstrated significantly greater cortical activation in the parietal association cortex under sham feedback conditions. Previous studies have suggested that the medial parietal association cortex is involved in memory-related visual imagery [57] and visuospatial imagery [58]. In addition, activation in the medial parietal association cortex is increased during visual imagery compared with kinesthetic imagery of body part movement [4]. Under sham conditions, the subjects could feel uncertain and lose confidence in kinesthetic imagery with incorrect feedback, which could mislead the subjects, because visual imagery would feel more familiar with healthy subjects and require less effort than kinesthetic imagery [59]. This could be responsible for enhanced activation in the medial parietal association cortex under sham conditions. In this study, subjects improved their kinesthetic motor imagery by trial-and-error. Under real feedback conditions, the feedback signals increased if the subjects activated motor-related cortex areas during the task condition and relaxed during the rest condition. This effect helped to learn how to perform proper kinesthetic imagery. However, that was not the case under sham feedback conditions; the subjects could feel unsure of their kinesthetic motor imagery. This could be responsible for the small and non-significant correlation between self-assessment scores of kinesthetic motor imagery and average feedback height in this study. A previous study using NIRS showed that positive and negative feedback increases motor imagery-related cortical activation [60], which suggests that it is not greater feedback, but rather appropriate feedback, of motor imagery performance that is most helpful for improved motor imagery. Further studies are needed to determine the most effective feedback method for improving behavioral performance.

Bilateral prefrontal cortex and right premotor cortex were activated by the motor imagery task, regardless of type of provided feedback (real or sham). Because motor imagery requires much attention and concentration, prefrontal activation could be related to cognitive processes involved in motor imagery. Indeed, previous reports have consistently documented greater cortical activation in the bilateral premotor cortex and prefrontal cortex during motor imagery tasks compared with motor execution tasks [3], [5].

Because cognitive processes, including planning, inhibition, and motor imagery, could evoke changes in heart and respiratory rate [12], [61], [62], it is possible that a systemic vascular response *via* the autonomic nervous system, which was evoked by motor imagery, could have affected the task-related hemoglobin signal changes. However, the focal activation patterns were different between feedback conditions and were unlikely to be the result of extracerebral contamination. Previous studies have introduced techniques to eliminate the effect of systemic vascular changes on NIRS signals [63], [64], [65], which should be adopted in the case of extracerebral signal contamination. Further development of systems to adopt these methodologies would help to improve task flexibility.

Sham information served as feedback information for the control condition. In previous neurofeedback studies, several kinds of signals, including background signals with random fMRI noise [29], signals from different brain lesions [27], signal from other subjects [27], and signals from different region in the previous session [66] were used as controls. The widespread cortical area includes the premotor and sensorimotor cortex, as well as the supplementary motor area, prefrontal cortex, and the parietal cortex [2], [5], [16], [67], [68]. In the present study, the NIRS system measured activation only from the fronto-parietal cortical area. Therefore, all available channels could have been activated by the motor imagery task. For this reason, the randomized value was utilized for control (sham) feedback.

### Limitations

There were several limitations in this study. First, because the study included only healthy subjects, the effect of neurofeedback on stroke patients remains unclear. Although motor imagery in stroke patients is generally not impaired [69], [70], the impairment level depends on site and extent of lesion [71], [72]. Accuracy and temporal coupling of motor imagery can be disrupted in some stroke patients, and further studies are needed to validate the effect of neurofeedback on motor imagery in stroke patients. Second, the long-term effect of neurofeedback on motor imagery and related cortical activation was not analyzed. A long-term effect of neurofeedback on motor imagery-related cortical activation of up to two weeks has been previously described in fMRI-mediated neurofeedback systems [66]. However, a NIRS-mediated system should be tested for long-term use in clinical applications. Third, differences in pace and complexity of imagery could have affected cortical activations between the two feedback conditions [34], [73]. Although the subjects were asked to imagine movement in a similar manner and at similar pace as was physically performed, it is possible that greater activation would result from faster or more complex finger imagery. Finally, only visual inspection was performed to detect overt finger movements during motor imagery without EMG monitoring. The subject finger was not constrained to avoid isometric muscle contraction, and subjects with overt finger movements were excluded. However, it could be possible that minimal muscle activation was evoked during imagery. Although marginal muscle activation was not excluded, it was assumed that these activities would not significantly differ between the feedback conditions. Comparison of timeline analysis and second-level imaging analysis under both feedback conditions revealed comparable activation in channels covering the sensorimotor cortex (channel 4). Previous results revealed that motor execution involves the limited area of the sensorimotor cortex [74]. Therefore, taking the poor spatial resolution of NIRS into consideration, it was assumed that the effect of subliminal EMG activation remained limited in this study.

In conclusion, results from the present study demonstrated the feasibility of a NIRS-mediated neurofeedback system and revealed the modulative effect of this system on motor imagery-related cortical activation. Results suggested that neurofeedback could facilitate individual skills for kinesthetic motor imagery. The NIRS-mediated neurofeedback system could be a promising tool, which could be applied in widespread areas, including neurorehabilitation. However, further clinical trials are needed to determine whether this system could enhance mental practice in stroke patients.
